# Total endovascular repair of a post-TEVAR Crawford extent II thoracoabdominal aortic aneurysm with concomitant renal artery aneurysm: a case report

**DOI:** 10.3389/fsurg.2026.1802270

**Published:** 2026-06-09

**Authors:** Xingbo Cao, Wei Liu, Miao Chen, Chi Cui

**Affiliations:** 1 North Sichuan Medical College, Nanchong, China; 2 Center of Vascular and Interventional Surgery, Department of General Surgery, The Third People's Hospital of Chengdu, Affiliated Hospital of Southwest Jiaotong University & The Second Affiliated Hospital of Chengdu, Chongqing Medical University, Chengdu, China

**Keywords:** 3D printing, physician-modified stent graft system, renal artery aneurysm, thoracoabdominal aortic aneurysm, total endovascular repair

## Abstract

Emergency management of post-dissection thoracoabdominal aortic aneurysm (pd-TAAA) in patients with complex anatomy, multiple comorbidities, and severely compromised physiological reserve presents a formidable challenge. Achieving accurate and safe endovascular rescue is extremely difficult, particularly in resource-limited environments where custom-made devices are not available. A 56-year-old male was admitted emergently with a giant Crawford extent II pd-TAAA measuring 71 mm in maximum diameter, complicated by a concomitant left renal artery aneurysm and reduced physiological reserve, including renal and hepatic insufficiency. Due to urgent symptoms and unavailability of off-the-shelf branched stent grafts, total endovascular repair was performed using a 3D-printed model-guided physician-modified stent graft system (PMSGS). This technique utilizes *in vitro* replication of the patient's anatomy to achieve accurate alignment between stent fenestrations and visceral artery ostia. Intraoperatively, a stent-assisted coil embolization strategy was applied to exclude the renal artery aneurysm, achieving “zero warm ischemia” protection for the kidney. Concurrently, a multimodal neuroprotective strategy, including the use of an iliac branch endoprosthesis and cerebrospinal fluid drainage, was utilized. Postoperative imaging confirmed complete aneurysm exclusion, patent visceral branches, and absence of spinal cord ischemia or renal function deterioration. This case elucidates that for pd-TAAA patients with complex anatomy and multiple high-risk factors in emergency settings, the 3D-printed model-guided PMSGS technique provides an accurate and effective alternative treatment strategy. Integration of this technique with comprehensive perioperative management efficiently solves anatomical matching difficulties and maximizes organ protection in the absence of custom-made devices.

## 1 Introduction

Thoracic endovascular aortic repair (TEVAR) is widely accepted as the first-line therapy for complicated type B aortic dissection (1). However, late failure of aortic remodeling poses a significant hindrance. Persistent false lumen patency driven by distal re-entry tears results in continued pressurization and extensive aneurysmal dilation (2). Managing post-dissection thoracoabdominal aortic aneurysm (pd-TAAA) accounts for one of the most challenging fields in vascular surgery. Although open surgery remains the gold standard for durable reconstruction (3), its associated high mortality and risk of postoperative renal failure limit its application in patients with pre-existing renal impairment and a high-risk clinical profile. Total endovascular repair utilizing off-the-shelf or custom-made devices has greatly enhanced the therapeutic landscape for pd-TAAA (4). However, in many medical centers globally, these advanced commercial devices are not yet regulatory-approved or immediately available. In such scenarios, particularly for high-risk patients who cannot wait for customized devices due to urgent symptoms, the physician-modified stent graft system (PMSGS) becomes a crucial, accessible, and cost-effective solution for complex anatomical reconstruction.

When a thoracoabdominal aortic aneurysm is complicated by visceral artery pathologies, such as a renal artery aneurysm, therapeutic complexity increases markedly. This demands not only accurate visceral vessel reconstruction but also the resolution of extremely challenging access conflicts. A highly challenging case of a giant Crawford extent II pd-TAAA was herein reported. The patient had undergone TEVAR 12 years prior, but extensive false lumen expansion occurred due to untreated distal re-entry tears. Due to the need for timely intervention as a result of sudden symptoms, pre-existing renal insufficiency, and the limited accessibility of commercial fenestrated grafts at the institution, the PMSGS technique was employed to concurrently repair the extensive aortic aneurysm and the concomitant left renal artery aneurysm. This case elucidates the feasibility and strategic planning essentials of PMSGS as a robust therapeutic strategy for managing such complex aortic pathologies in resource-limited environments.

## 2 Case presentation

A 56-year-old male presented to the emergency department with a sudden onset of persistent upper abdominal pain lasting approximately 10 h. He had undergone TEVAR for type B aortic dissection 12 years earlier; however, the distal re-entry tear in the abdominal aorta had not been addressed during the initial procedure. He had a history of hypertension and hyperlipidemia for over 10 years, as well as poorly controlled gout. He had been on chronic oral aspirin therapy. On physical examination, a palpable pulsatile mass measuring approximately 7 × 8 cm was detected in the lower abdomen. Neurological assessment indicated newly developed diminished superficial sensation in the lower limbs and reduced muscle strength, reduced to grade 4, suggesting potential spinal cord or limb nerve ischemia. Admission laboratory investigations demonstrated impaired overall physiological reserve, including renal insufficiency (serum creatinine 138 μmol/L [1.56 mg/dL], estimated glomerular filtration rate 52.41 mL/min/1.73 m^2^), hepatic dysfunction (total bilirubin 37.1 μmol/L, direct bilirubin 12.7 μmol/L, international normalized ratio 1.74), hypoalbuminemia (albumin 33 g/L), and moderate anemia (hemoglobin 87 g/L). Computed tomography angiography (CTA) indicated an appropriate morphology of the thoracic aortic stent graft. However, a giant post-dissection aneurysm was observed extending from the level of the left subclavian artery to the iliac bifurcation, with a maximum diameter of 71 mm. The abdominal aorta and left common iliac artery exhibited a persistent double-lumen sign. The dissection involved the ostia of multiple visceral branches, including the celiac axis, superior mesenteric artery, and bilateral renal arteries. The celiac axis and superior mesenteric artery were supplied by the true lumen, the right renal artery by the false lumen, and the left renal artery by both the true and false lumens. Additionally, a 2.03-cm saccular aneurysm was identified in the left renal artery, with the aneurysm neck located immediately adjacent to the vascular bifurcation (Figures 1, 2).

**Figure 1:**
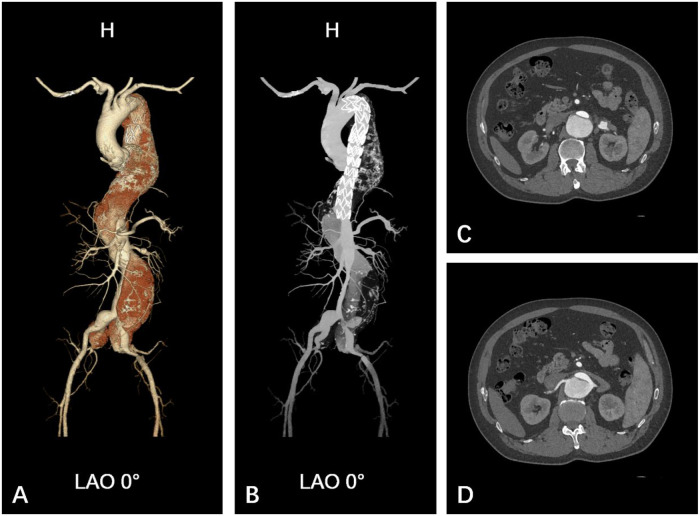
Preoperative CTA imaging. **(A)** 3D reconstruction illustrating the previous TEVAR stent and the distal giant, tortuous thoracoabdominal aortic aneurysm. **(B)** Maximum intensity projection image. **(C)** One of the abdominal aortic dissection re-entry tears. **(D)** Saccular aneurysm at the orifice of the left renal artery.

**Figure 2:**
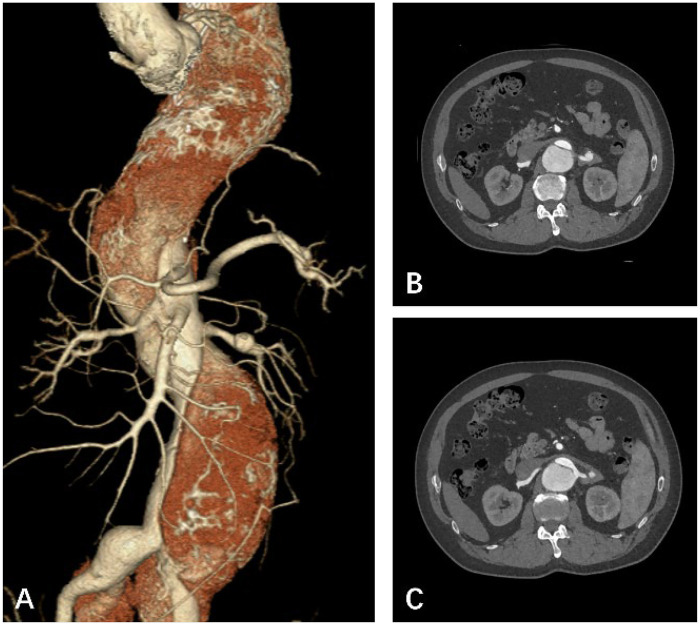
Preoperative imaging of the renal artery aneurysm. **(A)** Three-dimensional reconstruction showing the overall anatomical relationship of the left renal artery lesion. **(B,C)** Axial CTA images showing a focal saccular dilation in the proximal left renal artery. Based on preoperative CTA assessment, the aneurysm measured approximately 2.03 cm in maximum diameter and was located in the distal main trunk of the left renal artery adjacent to the bifurcation region. The left renal artery was supplied by both the true and false lumens.

Based on the acute presentation, the extensive disease burden, the complex anatomy, and objective evidence of reduced physiological reserve, including renal insufficiency, hypoalbuminemia, hepatic dysfunction, and moderate anemia, open repair was considered to carry a relatively high perioperative risk in this case. To minimize renal warm ischemia as much as possible, and considering that commercially available fenestrated or branched devices and custom-made devices were not available at our center, a decision was ultimately made, after full discussion with the patient and his family regarding the risks and benefits of different treatment strategies, to perform a single-stage total endovascular repair based on a physician-modified stent graft system. At the 6-month follow-up, the patient remained asymptomatic. Repeat CTA demonstrated patency of all visceral branches, complete exclusion of the aneurysm, and no endoleak or stent-related complications.

## 3 Operative technique

A cerebrospinal fluid drainage catheter was placed preoperatively to monitor and reduce intrathecal pressure. Intraoperative hemodynamic management followed a permissive hypertension strategy, maintaining a mean arterial pressure above 90 mmHg, alongside active correction of anemia to sustain hemoglobin levels above 100 g/L to optimize collateral oxygen delivery and ameliorate the risk of spinal cord ischemia.

Under general anesthesia, bilateral brachial and femoral artery accesses were established. 18F and 16F sheaths were inserted into the right and left femoral arteries, respectively. A patient-specific 3D-printed aortic model (transparent photosensitive resin) was generated preoperatively based on the patient's CTA data. On the sterile field, a 28–120 mm straight aortic covered stent graft (Lifetech Scientific, Shenzhen, China) was partially deployed within the 3D-printed model, and alignment markings were made according to the spatial positions of the ostia of the celiac axis, superior mesenteric artery, and bilateral renal arteries (Figure 3). Subsequently, four fenestrations were created using a medical electric cautery pen (CIRX, Inc, Ningbo, China), and the edges of the fenestrations were reinforced with platinum coils (Cook Medical, Bloomington, IN, USA), which also served as radiopaque markers during the procedure. After modification, the stent graft was re-constrained using a V18 guidewire (Boston Scientific, Marlborough, MA, USA) with the diameter-reducing tie technique, and was then reloaded into the delivery system.

**Figure 3:**
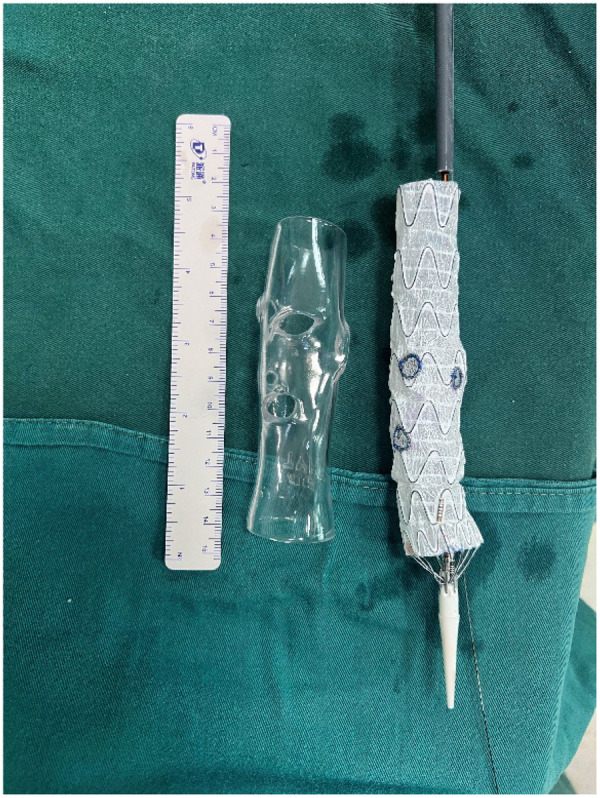
3D-printed model and PMSGS.

Main body deployment and visceral branch reconstruction were performed after delivery of the PMSGS through the right femoral artery. Under fluoroscopic guidance, the device was gradually deployed and precisely aligned with the fenestration markers corresponding to the celiac axis, superior mesenteric artery, and bilateral renal arteries. Intraoperative angiography confirmed that the celiac axis and superior mesenteric artery were supplied by the true lumen, the right renal artery by the false lumen, and the left renal artery by both the true and false lumens. Visceral branch reconstruction was accomplished using covered stents (W. L. Gore & Associates, Flagstaff, AZ, USA) to bridge the fenestrations. The right renal artery was superselected via the brachial approach. Access to the false lumen–supplied right renal artery was established through a pre-existing re-entry tear combined with septal fenestration, after which balloon dilation was performed at the corresponding fenestration and a 7 × 50 mm stent graft was implanted. The superior mesenteric artery was then superselected via the brachial approach, followed by fenestration-level balloon dilation and implantation of an 8 × 25 mm stent graft. The celiac axis was cannulated through the left femoral approach, and an 8 × 25 mm stent graft was implanted. After deployment of all bridging covered stents, balloon-expandable stents (Abbott Vascular, Santa Clara, CA, USA) were further implanted to reinforce each branch, thereby enhancing radial support at the fenestration sites and improving stent apposition.

Preoperatively, the left renal artery exhibited dual perfusion from both the true and false lumens, and reconstruction was ultimately performed through the true lumen during the procedure. Via the left femoral approach, the left renal artery ostium was superselected using a guidewire-catheter system. To simultaneously reconstruct the main trunk of the left renal artery and treat the concomitant aneurysm, a dual-guidewire branch cannulation strategy was adopted. One guidewire was used to establish support access to the main trunk of the left renal artery and was then exchanged for a stiff guidewire to facilitate delivery of the covered stent. The other guidewire was exchanged for a microcatheter and advanced further into a distal branch of the left renal artery, thereby preserving access to the aneurysm sac. Over the stiff guidewire, a 7 × 25 mm covered stent (GORE® VIABAHN® endoprosthesis, W. L. Gore & Associates, Flagstaff, AZ, USA) was deployed across the segment adjacent to the left renal artery aneurysm to reconstruct flow in the main trunk of the left renal artery, while the microcatheter was maintained outside the stent graft and directed into the aneurysm sac. Detachable coils were then delivered into the aneurysm sac through the microcatheter until angiography confirmed satisfactory embolization. After completion of sac embolization, the microcatheter was withdrawn, and a second 7 × 25 mm covered stent was deployed in an overlapping stent-in-stent configuration with the first stent to further seal the aneurysm neck and enhance local sealing. The proximal end of the second stent protruded approximately 3 mm into the aortic lumen, and its distal end was deployed within the left renal artery in overlap with the first stent. Final angiography demonstrated patency of the main trunk of the left renal artery, no further opacification of the aneurysm sac, and preserved perfusion of the distal renal parenchyma.

Intraoperative angiography demonstrated a type Ia endoleak, which was successfully treated with the deployment of a 30 mm diameter aortic cuff (Lifetech Scientific), which was implanted proximally. Distally, a 28 mm diameter bifurcated aortic stent graft (Lifetech Scientific) was implanted to connect the PMSGS to the iliac system, with proximal overlap. The right internal iliac artery (IIA) was embolized, while the left iliac system was reconstructed using an Ankura iliac branch endoprosthesis (Lifetech Scientific) to preserve the left IIA. Final angiography confirmed the desired exclusion of the aneurysm and patency of all visceral and branch vessels (Figure 4).

**Figure 4:**
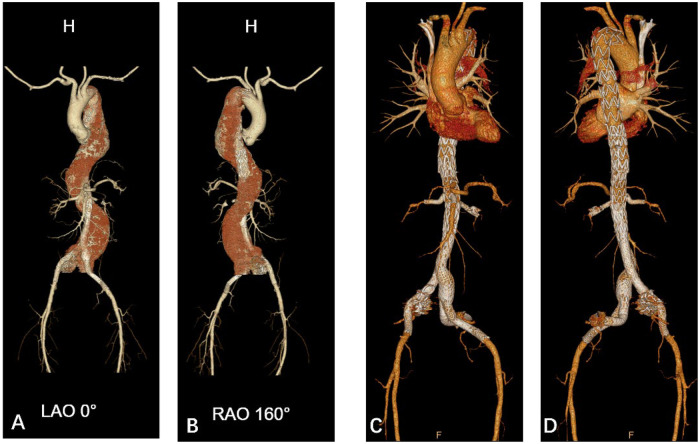
Postoperative CTA imaging. **(A,B)** Images at 4 days post-operation. **(C,D)** Follow-up images at 6 months post-operation.

## 4 Discussion

This case report describes an extremely challenging clinical scenario of chronic post-dissection thoracoabdominal aortic aneurysm after previous TEVAR. The complexity of this case was attributable not only to the extensive disease burden, but also to the highly complex true- and false-lumen perfusion patterns of the visceral branches and the concomitant left renal artery aneurysm, all of which further increased the technical difficulty of visceral branch reconstruction and organ protection. For Crawford extent II lesions of this type, open repair remains an important benchmark in terms of long-term durability (3). In selected high-risk patients, staged or hybrid repair may also be considered to reduce the burden of a single procedure and mitigate the risk of spinal cord ischemia (4). Previous reports have suggested that open conversion after TEVAR may, in some cases, even provide favorable technical conditions for distal open clamping (5). However, open conversion after TEVAR is itself a highly complex aortic procedure, and its perioperative morbidity and mortality are not negligible; the actual risk is influenced by multiple factors, including the extent of the lesion, the clamping strategy, the patient's baseline condition, and institutional experience. In the present case, the patient presented with acute symptom onset and reduced physiological reserve, both of which predicted a higher perioperative risk for open repair. If a staged or hybrid strategy had been pursued, it might theoretically have reduced the burden of a single procedure; however, the dissection-related aneurysm still carried a potential risk of further progression, symptom worsening, or even rupture. In addition, the left renal artery aneurysm required concomitant treatment, and once the main stent graft and left renal artery bridging reconstruction had been completed, subsequent re-access to the aneurysm sac would have been markedly restricted. By contrast, a single-stage total endovascular repair allowed simultaneous exclusion of the lesion, reconstruction of the visceral branches, and concomitant treatment of the left renal artery aneurysm in one procedure, while minimizing additional renal warm ischemia as much as possible and avoiding the additional time and financial burden associated with staged treatment. Given that commercially available fenestrated or branched devices, as well as custom-made devices, were not available at our center, the final decision to perform a single-stage total endovascular repair based on the PMSGS technique represented a pragmatic choice after comprehensive consideration of the procedural risks, anatomical characteristics, and resource constraints in this case (6, 7). It should be noted that, under non-urgent or elective conditions and provided that the patient's physiological status permits, open repair as well as staged/hybrid repair should also be included among the alternative treatment options.

Because of the long-standing chronic dissection, the aortic true lumen was markedly narrowed and tortuous, and the spatial relationship among the visceral artery ostia was highly complex, making accurate fenestration planning difficult when relying on CTA-based vascular modeling alone (8). In this context, the 3D-printed model served as a supplement to standard CTA-based preoperative planning. By allowing *in vitro* deployment of the main stent graft and fenestration positioning according to the individualized spatial relationship of the visceral branch ostia, it helped improve the accuracy of customized fenestration design. In addition, the model provided an intuitive representation of the patient's complex dissected anatomy, facilitating the operator's understanding of the three-dimensional spatial relationship among multiple branch ostia within the narrowed true lumen. Although the patient had an acute onset of symptoms, the 3D model based on CTA data could be completed at our center within 3–6 h. While this approach is not suitable for true emergency cases with rupture or hemodynamic instability requiring immediate intervention, it remains realistically feasible for urgent cases such as the present one, in which a limited preoperative preparation window is available. However, no direct comparison with conventional 2D measurement-based modification was performed in this case; therefore, whether the 3D-printed model reduced operative time or fluoroscopy time remains uncertain.

A critical technical decision in this case was the simultaneous exclusion of the left renal artery aneurysm (9). Once the PMSGS main body is deployed, its fabric spans the renal artery ostium. Although blood flow is reconstructed through the fenestration, the stent structure physically blocks catheter access to the aneurysm sac, effectively precluding a pathway for future endovascular intervention. For this patient with renal insufficiency and a baseline creatinine of 138 μmol/L, any form of warm ischemia could lead to irreversible nephron loss (10). The total endovascular technique, performed through a microcatheter in the stent interstices, achieved dense packing while maintaining continuous renal perfusion, effectively realizing a “zero warm ischemia” procedure and maximizing preservation of the patient's residual renal function.

Although this case involved extensive coverage from the thoracic aorta to the iliac arteries, which is typically considered a high-risk factor for paraplegia (11–13), no symptoms of spinal cord ischemia postoperatively were developed by the patient. This satisfactory outcome was attributed to the implementation of a multimodal neuroprotective strategy. First, the staged repair played a foundational protective role. The TEVAR performed 12 years prior served as the first stage, promoting proliferation and remodeling of the paraspinal collateral network, thereby enhancing spinal cord tolerance to the current extensive coverage (14). Second, the preservation of pelvic blood flow was given extreme importance. According to Griepp's collateral network concept (14), the IIA is a vital blood supply source for the conus medullaris and lower spinal cord. Consequently, bilateral IIAs were not simply embolized; instead, the iliac branch endoprosthesis technique was employed to reconstruct the left IIA, maximizing collateral perfusion from the pelvis to the spinal cord. Finally, strict perioperative adjunctive measures constituted the last line of defense. A prophylactic cerebrospinal fluid drainage catheter was placed preoperatively to reduce intrathecal pressure, and active hemodynamic management, including permissive hypertension (MAP > 90 mmHg) and active correction of preoperative anemia (Hb > 100 g/L), to optimize oxygen delivery to the spinal cord through the collateral network (4).

However, there are certain limitations in the study. As a single case report, the evidence level is limited, and the generalizability of the conclusions requires verification through larger cohort studies. The PMSGS technique involves the “off-label” modification of commercial stent grafts. Although early results are satisfactory, potential concerns regarding long-term structural integrity, specifically the risk of suture breakage or fabric fatigue, remain unexplored. Consequently, strict long-term imaging follow-up for this patient is mandatory to monitor for the occurrence of late complications.

In summary, this case confirms that in resource-limited settings lacking custom-made devices, the 3D-printed model-guided PMSGS technique is a realistic and efficient therapeutic strategy for Crawford extent II pd-TAAA patients with complex anatomy and reduced physiological reserve. The success of this procedure relies not only on high-precision stent modification techniques but also on a multimodal perioperative management strategy and bundled spinal cord protection measures. The experience provides a valuable clinical reference for performing total endovascular repair under extremely challenging anatomical and physiological conditions.

## Data Availability

The original contributions presented in the study are included in the article/Supplementary Material, further inquiries can be directed to the corresponding author.
