# Supplementary material for: Total endovascular repair of a post-TEVAR Crawford extent II thoracoabdominal aortic aneurysm with concomitant renal artery aneurysm: a case report
Source: Front Surg. 2026 Jun 9;13:1802270. doi: 10.3389/fsurg.2026.1802270 (PMC13286951; doi:10.3389/fsurg.2026.1802270)
Supplement: Supplementary file 1 [file Presentation1.pptx]

## Slide 1
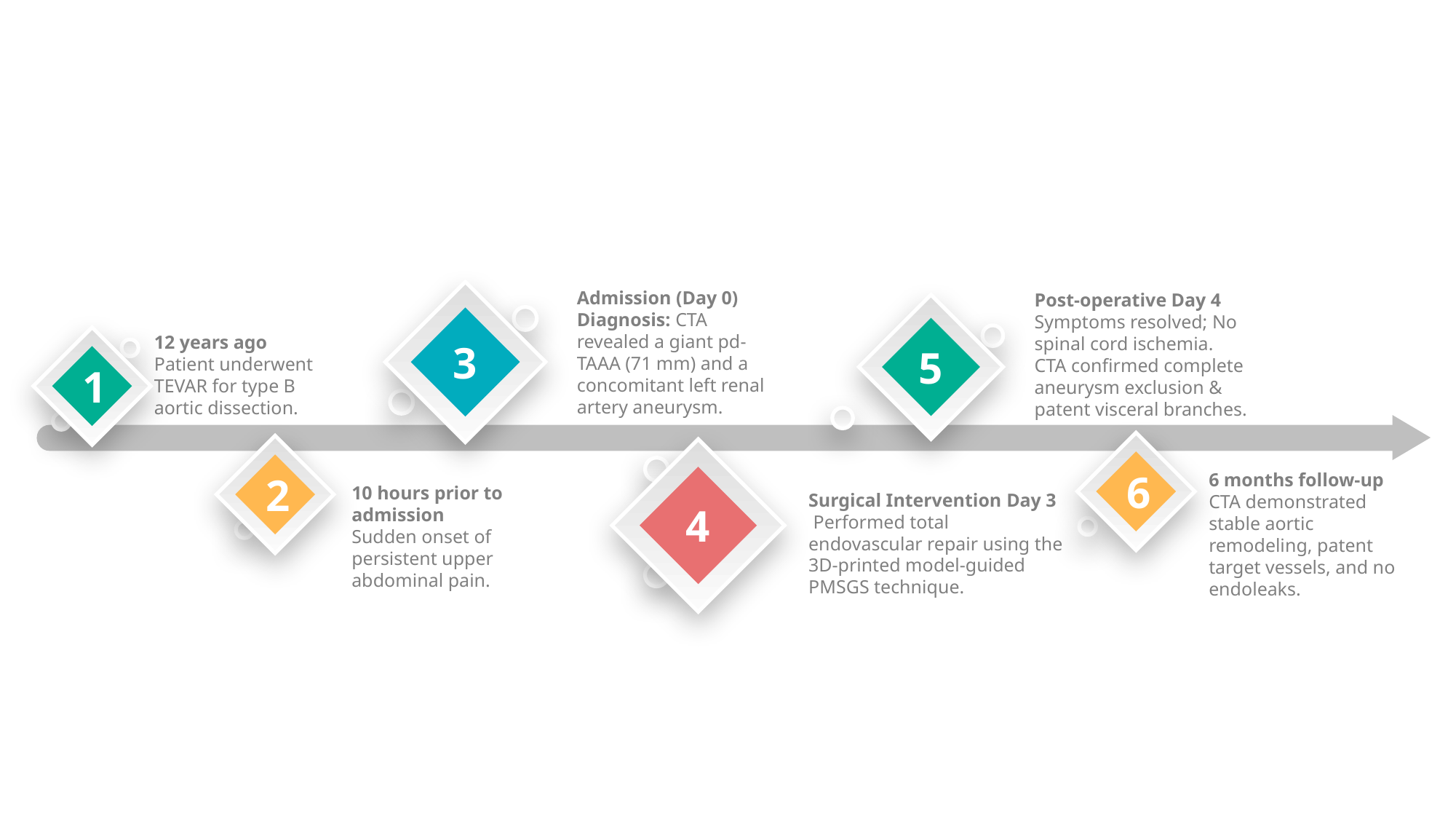

3
Admission (Day 0) Diagnosis: CTA revealed a giant pd-TAAA (71 mm) and a concomitant left renal artery aneurysm.
Post-operative Day 4
Symptoms resolved; No spinal cord ischemia.
CTA confirmed complete aneurysm exclusion & patent visceral branches.
5
12 years ago
Patient underwent TEVAR for type B aortic dissection.
1
6
2
4
6 months follow-up
CTA demonstrated stable aortic remodeling, patent target vessels, and no endoleaks.
10 hours prior to admission
Sudden onset of persistent upper abdominal pain.
Surgical Intervention Day 3
 Performed total endovascular repair using the 3D-printed model-guided PMSGS technique.
